# CSF levels of brain-derived proteins correlate with brain ventricular volume in cognitively healthy 70-year-olds

**DOI:** 10.1186/s12014-024-09517-1

**Published:** 2024-12-12

**Authors:** Sofia Bergström, Sára Mravinacová, Olof Lindberg, Anna Zettergren, Eric Westman, Lars-Olof Wahlund, Kaj Blennow, Henrik Zetterberg, Silke Kern, Ingmar Skoog, Anna Månberg

**Affiliations:** 1 Division of Affinity Proteomics, Department of Protein Science, KTH Royal Institute of Technology, SciLifeLab, Stockholm, Sweden; 2 Division of Clinical Geriatrics, Department of Neurobiology, Center for Alzheimer Research, Care Sciences and Society, Karolinska Institutet, Stockholm, Sweden; 3 Neuropsychiatric Epidemiology Unit, Department of Psychiatry and Neurochemistry, Institute of Neuroscience and Physiology, Sahlgrenska Academy, Centre for Ageing and Health (AGECAP), University of Gothenburg, Mölndal, Sweden; 4 Clinical Neurochemistry Laboratory, Sahlgrenska University Hospital, Mölndal, Sweden; 5 Department of Psychiatry and Neurochemistry, Institute of Neuroscience and Physiology, The Sahlgrenska Academy at the University of Gothenburg, Mölndal, Sweden; 6 UK Dementia Research Institute at UCL, London, UK; 7 Department of Neurodegenerative Disease, UCL Institute of Neurology, London, UK; 8 Hong Kong Center for Neurodegenerative Diseases, Clear Water Bay, Hong Kong China; 9 Wisconsin Alzheimer’s Disease Research Center, University of Wisconsin, School of Medicine and Public Health, University of Wisconsin-Madison, Madison, WI USA; 10 Region Västra Götaland, Sahlgrenska University Hospital, Neuropsychiatry, Mölndal, Sweden

**Keywords:** Protein profiling, Cerebrospinal fluid, Brain ventricular volume, Cortical thickness, Diffusion tensor imaging, Brain-enriched proteins, Inter-individual variability

## Abstract

**Background:**

The effect of varying brain ventricular volume on the cerebrospinal fluid (CSF) proteome has been discussed as possible confounding factors in comparative protein level analyses. However, the relationship between CSF volume and protein levels remains largely unexplored. Moreover, the few existing studies provide conflicting findings, indicating the need for further research.

**Methods:**

Here, we explored the association between levels of 88 pre-selected CSF proteins and ventricular volume derived from magnetic resonance imaging (MRI) measurements in 157 cognitively healthy 70-year-olds from the H70 Gothenburg Birth Cohort Studies, including individuals with and without pathological levels of Alzheimer’s disease (AD) CSF markers (*n* = 123 and 34, respectively). Both left and right lateral, the inferior horn as well as the third and the fourth ventricular volumes were measured. Different antibody-based methods were employed for the protein measurements, with most being analyzed using a multiplex bead-based microarray technology. Furthermore, the associations between the protein levels and cortical thickness, fractional anisotropy, and mean diffusivity were assessed.

**Results:**

CSF levels of many brain-derived proteins correlated with ventricular volumes in A-T- individuals, with lower levels in individuals with larger ventricles. The strongest negative correlations with total ventricular volume were observed for neurocan (NCAN) and neurosecretory protein VGF (rho = -0.34 for both). Significant negative correlations were observed also for amyloid beta (Ab) 38, Ab40, total tau (t-tau), and phosphorylated tau (p-tau), with correlation ranging between − 0.34 and − 0.28, while no association was observed between ventricular volumes and Ab42 or neurofilament light chain (NfL). Proteins with negative correlations to ventricular volumes further demonstrated negative correlations to mean diffusivity and positive correlation to fractional anisotropy. However, only weak or no correlations were observed between the CSF protein levels and cortical thickness. A + T + individuals demonstrated higher CSF protein levels compared to A-T- individuals with the most significant differences observed for neurogranin (NRGN) and synuclein beta (SNCB).

**Conclusions:**

Our findings suggest that the levels of many brain-derived proteins in CSF may be subjected to dilution effects depending on the size of the brain ventricles in healthy individuals without AD pathology. This phenomenon could potentially contribute to the inter-individual variations observed in CSF proteomic studies.

**Supplementary Information:**

The online version contains supplementary material available at 10.1186/s12014-024-09517-1.

## Introduction

The cerebrospinal fluid (CSF) proteome is a useful source of information for both diagnostic and research purposes in the context of neurological disorders. It contains a wide range of proteins originating both from the periphery and the central nervous system. From numerous studies analysing protein levels in relation to neurodegenerative disorders, it has become apparent that the individual variation is large and that proteins identified as disease-associated in group comparisons, although significantly different, often overlap substantially. This overlap limits the potential use of protein levels as clinical biomarkers and the cause of variation among individuals is so far not fully understood.

In adults, the CSF volume is traditionally considered approximately 150 ml, distributed as 125 ml in the subarachnoid space and 25 ml in the ventricles [1]. Recent data however indicate that these volumes might be underestimations with new estimates of total volumes exceeding 200 ml [2, 3]. CSF volume also varies among individuals and is strongly associated with total intracranial volume (ICV) and sex, with both higher CSF volume and ICV in males [3–5]. Furthermore, both CSF volume and overall brain anatomy have in several studies been shown to associate with specific genetic variations such as in the genomic GMNC-OSTN region [6, 7]. While sex and genetics represent static explanations of individual variation, there are other factors with more dynamic impact, possibly explaining why this variation has been seen to increase during life [8]. A substantial increase in intracranial CSF volume is suggested to occur at older ages according to both cross-sectional [3, 4, 8, 9] and longitudinal [10] studies. For example, Yamada and colleagues reported the intracranial CSF volume to double in individuals without neurological symptoms from the 20s (265 ml) to the 80s (488 ml). The increase in the subarachnoid space followed a linear trend while ventricular volumes increased after the age of 60 [3]. The longitudinal study design by Jochems et al. illustrates both the age-associated increase of ventricular volume and the individual variation at specific ages [10]. In addition to what appear to be age-related processes independent of pathological conditions, there is also a number of diseases that can influence CSF volume through extensive atrophy such as in Alzheimer’s disease (AD) and impaired CSF flow as seen in idiopathic normal pressure hydrocephalus (iNPH) patients [2].

The effect of varying CSF volume on the CSF proteome has so far not been widely studied. It can be speculated that individuals with higher volumes caused by other reasons than large ICV would display generally lower protein levels due to a higher degree of dilution. If so, this could be a potential confounder in all analysis comparing protein levels between individuals, especially in the elderly population. Some studies have investigated the association between CSF levels of amyloid beta (Aβ) peptides and tau proteins with ventricular volume in healthy [11] and iNPH patients [5]. Neither of these studies found any association with total tau (t-tau) or phosphorylated tau (p-tau) levels but a negative correlation between different Aβ peptides and ventricular volume. However, a third study including AD patients found a negative correlation also for tau in the AD and mild cognitive impairment (MCI) patient population but not in healthy individuals [12]. Others have investigated the associations between these proteins and choroid plexus volume and reported negative correlations in amyloid negative but not amyloid positive individuals [13]. In this particular study, significant associations were not seen for ventricular volumes, however choroid plexus and ventricular volume did correlate positively. A recent study by Hansson and colleagues investigated the impact of genetic regulation on the CSF proteome and found, in addition to a number of proteins affected by genetic variants, that levels of many brain-derived proteins were associated with ventricular volumes [6].

As the associations between CSF protein levels and brain ventricular volumes have not been extensively studied and the existing results are conflicting, the aim of our work was to investigate these associations in more detail.

## Materials and methods

### Sample cohort

The participants included in the presented study represents a subset of individuals from the Birth cohort 1944 in the multidisciplinary epidemiological H70 Gothenburg Birth Cohort Studies [14]. Based on the Swedish Population Registry, all individuals living in Gothenburg, Sweden born on prespecified dates during 1944 were invited to participate in the studies and the examinations took place 2014–2016. The response rate of the Birth cohort 1944 was 72% and 1203 individuals participated [15]. Among these, 277 underwent both lumbar puncture and magnetic resonance imaging (MRI). Six individuals with iNPH diagnosis or probable iNPH based on radiological examination were excluded from the dataset. The study was approved by the Regional Ethics Review Board in Gothenburg (Approval Numbers: 869 − 13, 006–14, and T703-14) and all participant and/or their close relatives gave written informed consent to participate in the study.

Extensive clinical cognitive examinations were performed including, among others, rating based on Clinical Dementia Rating (CDR) [16] and Mini-Mental State Examination (MMSE) [17]. The majority of the 277 individuals with available CSF sample and MRI scan had a CDR score of zero (81%), but 51 individuals had a score of 0.5 and one individual had a score of 1. More details about the cognitive battery are described previously [14]. *APOE* genotyping was performed with the KASPar PCR SNP genotyping system (LGC Genomics, Hoddesdon, Herts, UK). *APOE*-ε4 status was defined as positive (ε2/ε4, ε3/ε4, ε4/ε4) or negative (ε2/ε2, ε2/ε3, ε3/ε3).

CSF was collected by lumbar puncture according to the standardized protocol within the H70 studies [18]. The CSF Aβ42/Aβ40 ratio was measured using the V-PLEX Aβ Peptide Panel 1 (6E10) Kit (Meso Scale Discovery) as described previously [14, 15]. The concentrations of CSF t-tau, p-tau (phosphorylated at threonine 181), and Aβ42 were determined using Enzyme-linked immunosorbent assays (ELISA) (INNOTEST). The analysis of these assays was performed as a part of the clinical routines at the Mölndal Clinical Neurochemistry laboratory. Individuals with an Aβ42/Aβ40 ratio below 0.082 were denoted amyloid-positive and the rest amyloid-negative. The cut-off value for pathological Aβ42/Aβ40 ratio was determined by the bimodal cut-point for all individuals with measured Aβ42/Aβ40 ratio (*n* = 318). Pathological levels of t-tau were defined as > 350 pg/ml, p-tau > 80 pg/ml and neurofilament light chain (NfL) > 1850 pg/ml. The CSF and serum albumin concentrations were measured by immunonephelometry on a Beckman Image Immunochemistry system (Beckman Instruments, Beckman Coulter) and the albumin quotient was calculated as CSF albumin (mg/l) over serum albumin (g/l).

For the purpose of this study, we only included individuals that fulfilled the criteria for one of the two following groups (Table 1). The first group (*n* = 123), denoted as A-T-, contained individuals exhibiting non-pathological Aβ42/Aβ40 ratio, t-tau levels, p-tau levels, and NfL levels, with no discernible cognitive impairment, defined as CDR = 0. The second group (*n* = 34), named A + T+, consisted of individuals with both pathological Aβ42/Aβ40 ratios and t-tau levels.

### Magnetic resonance imaging acquisition and analysis

The MRI data acquisition was performed using one 3.0 T Philips Achieva system (Philips Medical Systems), following the protocol standardized for the H70 studies. FreeSurfer 7.2, an open-source software package for processing and analyzing structural neuroimaging data, was used to assess brain volumes from the T1-weighted images [19, 20]. Reconstructed data sets were visually inspected for accuracy followed by calculations of ICV as well as different regions of interest. This study included measurements of the left and right lateral ventricles (both total volume and a specific measurement of the interior horns), as well as the third and fourth ventricles. The total ventricular volume was calculated as the sum of the lateral ventricles, the third ventricle, and the fourth ventricle. Additionally, the cortical thickness per hemisphere was determined. MRI data management and processing were performed with our database system, theHiveDB [21].

**Table 1:** Sample demographics. Continuous variables are presented in the format median [range]. P-values were obtained by Wilcoxon rank sum test and Fisher’s exact test, for numerical and categorical variables, respectively.

	A-T-	A + T+	*p*-value
n	123	34	
Age	71 [70–72]	71 [70–72]	0.81
Sex (F/M)	66/57	12/22	0.08
CDR (0/0.5)	123/0	27/7	1e-5
t-tau (pg/ml)	262 [115–349]	468 [354–963]	5e-19
p-tau (pg/ml)	39 [18–61]	68 [49–128]	1e-17
Aβ38 (pg/ml)	2269 [971–3461]	2981 [2127–4165]	2e-9
Aβ40 (pg/ml)	5848 [2752–7527]	7120 [5359–9760]	3e-9
Aβ42 (pg/ml)	770 [409–1140]	436 [195–628]	3e-16
Aβ42/Aβ40 ratio	0.139 [0.083–0.210]	0.061 [0.034–0.079]	5e-19
NfL (pg/ml)	651 [276–1829]	857 [483–1256]	6e-4
MMSE	30 [25–30]	29 [26–30]	5e-3
*APOE*-ε4 status (0/1/NA)	92/30/1	11/22/1	2e-5

Diffusion tensor imaging (DTI) was used to assess the microstructural white matter integrity. Two variables were included to assess the molecular displacement of water molecules by diffusion; mean diffusivity reflects the average magnitude whereas the fractional anisotropy reflects the directionality. Processing of diffusion-weighted images have previously been described [22, 23]. In brief, images were analyzed using the FMRIB’s Diffusion Toolbox from FSL (https://fsl.fmrib.ox.ac.uk/fsl/fslwiki) [24]. First, the data was corrected for eddy current distortion and head motion using the b0 non-diffusion data as a reference volume [25]. The resulting images were skull-striped and a diffusion tensor model was fitted at each voxel to determine the preferred diffusion direction as the principal eigenvector of the eigenvalue decomposition [26, 27]. To provide information on the microstructural organization of the white matter, the fractional anisotropy and mean diffusivity maps were computed for each voxel [28]. The fractional anisotropy maps were transformed into Montreal Neurological Institute (MNI) space using the tract-based spatial statistics tool. After normalization, fractional anisotropy images were resampled and subsequently merged into a single file to create a mean fractional anisotropy image for all subjects, which was then used to create a mean fractional anisotropy ‘skeleton’. The threshold of the skeleton was set to 0.2 to include the white matter tracts that were common to all subjects. Individual fractional anisotropy maps were subsequently projected onto this mean fractional anisotropy skeleton. The transformation matrix of fractional anisotropy obtained in the above steps was applied to mean diffusivity maps. Mean fractional anisotropy and mean diffusivity for each subject were extracted under the mean skeleton created for all subjects.

### Cerebrospinal fluid protein profiling

In-house developed ELISA assays were used to measure the concentrations of NfL [29] and neurogranin (NRGN) [30]. The CSF levels of 81 additional proteins (Supplementary Table 1) were measured using an antibody-based suspension bead array [31]. The proteins were selected based on their association with neurodegenerative diseases according to our other studies [32–36]. Many of the selected proteins show elevated expression levels in brain compared to other tissue types according to RNA expression data in the Human Protein Atlas (www.proteinatlas.org, version 24). A detailed description of the suspension bead array analysis was previously published [31]. In brief, polyclonal rabbit antibodies were selected from the available antibody set generated within the Human Protein Atlas and immobilized onto color-coded magnetic beads (MagPlex-C, Luminex Corporation) using carbodiimide crosslinker chemistry and was subsequently pooled to form the suspension bead array. The protein content of the CSF samples was directly labeled with a tenfold molar excess of biotin. Subsequently, the bead array was incubated with the biotinylated, diluted, and heat-treated CSF samples and read-out was facilitated using a streptavidin-coupled fluorophore in a FLEXMAP 3D instrument (Luminex Corporation). Results from the protein profiling were previously published with a focus on the association between these CSF protein levels and tau and Aβ [37].

### Data analysis

The open-source software R (version 4.3.2) [38] was used for data processing, statistical analysis, and visualizations. The data from the suspension bead array was normalized to diminish the effect of time delay during read-out using robust linear regression (MASS::rlm). The obtained residuals were added to the median signal intensity per protein. Subsequently, the potential differences between plates were reduced (limma:: removeBatchEffect).

To adjust for individual differences in head size, ventricular volumes were normalized using the residual approach based on the estimated total ICV [39]. To capture the “normal” relationship between the volume of interest and ICV, a robust linear regression was used to determine the slope $\:\beta\:$ of the regression line among the individuals in the A-T- group. In case of pathology this relationship might not necessarily be sustained, therefore the determined relationship in the healthy individuals was used to adjust the ventricular volumes in the remaining individuals as well.

No correction for age was conducted in the analysis since all participants were 70-year-olds (70–72 years). Spearman correlations between all protein levels and ventricular volumes were calculated and visualized in a heatmap (pheatmap::pheatmap) per group of individuals and annotated with the correlation to total ventricular volume and albumin quotient. The proteins were clustered based on the Euclidean distance using Ward’s clustering (ward.D2) and divided into two clusters (stats::cutree). The potential univariate differences of protein levels and MRI data between the different sample groups (A-T- vs. A + T+) were evaluated using Wilcoxon rank sum test. For transparency, results based on raw brain metrics, without adjustments for ICV, are included when suitable. A similar approach was conducted when assessing the correlation between protein levels and cortical thickness, diffusivity as well as fractional anisotropy.

Finally, to assess whether adjusting for ventricular volume would increase the separation between the sample groups, the protein levels were adjusted based on total ventricular volume using the same approach as when adjusting ventricular volumes based on ICV.

## Results

We investigated brain ventricular volumes as a potential confounder of CSF protein profiles in 157 70-year-olds. Study participants were split into amyloid and tau negative (A-T-, *n* = 123) vs. positive (A + T+, *n* = 34) based on Ab42/40 ratio and t-tau levels with the aim to explore the associations in individuals with and without early AD pathology.

### Brain ventricular volumes are associated with sex but not early AD pathology

**Fig. 1:**
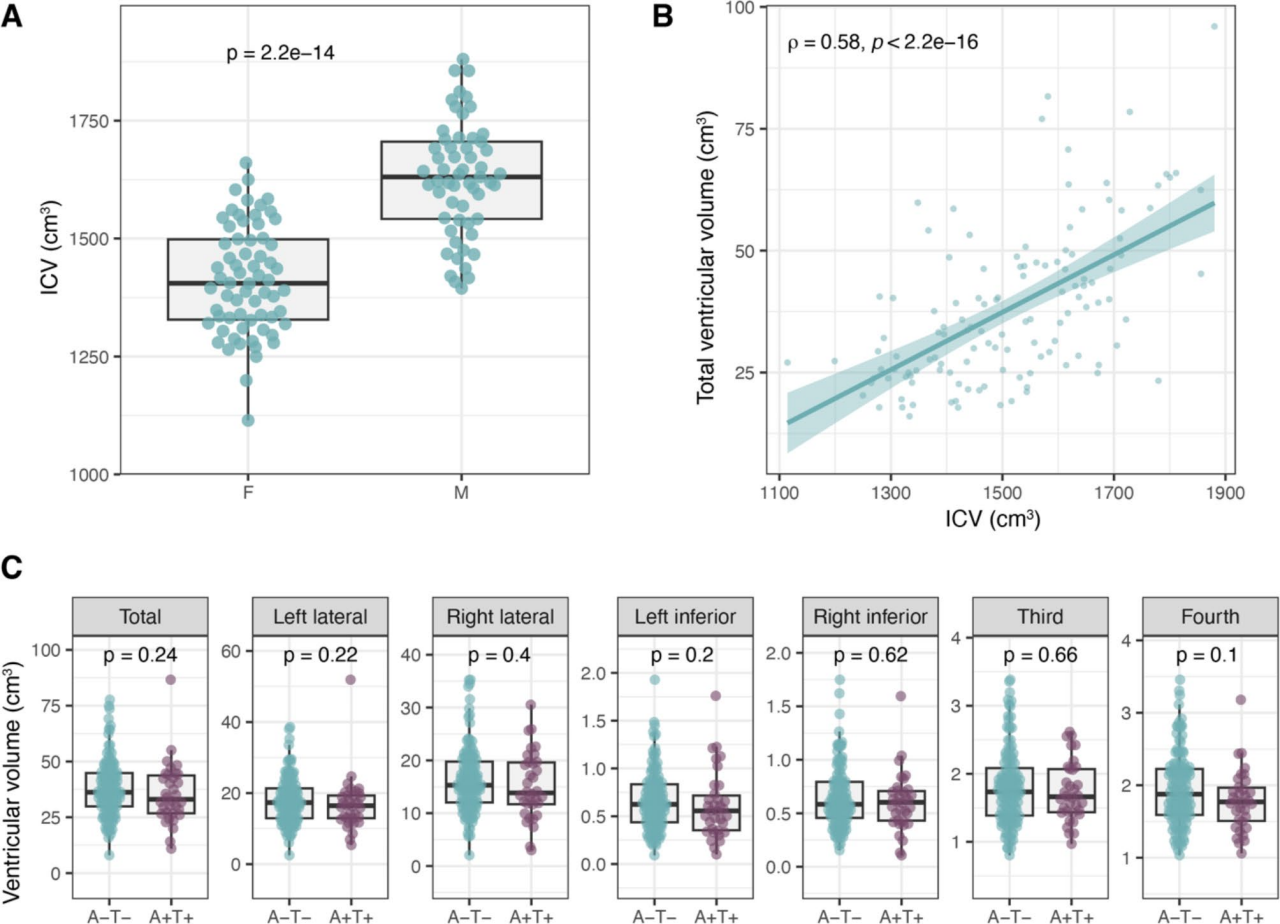
Ventricular volumes are similar in individuals with and without early AD pathology. **A**) Larger intracranial volume (ICV) was observed in males compared to females. **B**) Total ventricular volume correlates with ICV. **C**) No differences in ventricular volumes were observed between A-T- and A + T+, based on ICV adjusted volumes

Ventricular volumes were estimated using MRI imaging, with data for the left and right lateral, left and right inferior lateral horns, third, and fourth ventricles included in the analysis. Summarising data for the 123 A-T- individuals, volumes for all ventricles varied greatly as exemplified by the left lateral ventricle ranging from 6.9 to 45 ml (median 16 ml) (see Supplementary Table 2 for all volumes). Total ventricular volume was calculated as the sum of the lateral (both hemispheres), third and fourth ventricles and ranged between 16 and 96 ml (median 34 ml). Comparing ventricular volumes among A-T- individuals, left and right lateral ventricles showed strong positive correlation to each other (rho = 0.91), as did the left and right inferior horns of the lateral ventricles (rho = 0.79) while the volumes of the fourth ventricle were most deviating from the others (rho ranging from 0.35 to 0.45) (Supplementary Fig. 1A). All ventricular volume measures, except the fourth ventricle, showed significant sex differences within the healthy group (A-T-) with larger volumes in males than females (Supplementary Fig. 1B), mirroring the results for intracranial volume (ICV) (*p* = 2.2 × 10^− 14^) (Fig. 1A).

Among the A-T- individuals, males had 16% larger ICV compared to females. Furthermore, all ventricular volumes correlated positively to ICV (rho ranging from 0.27 to 0.59) (Fig. 1B and Supplementary Fig. 2A). To account for the impact of ICV on ventricular volumes in subsequent analysis we adjusted the volumes using robust linear regression. The ICV adjusted data showed no significant association with sex (Supplementary Fig. 2B) nor difference in ventricular volumes between A-T- and A + T + groups (Fig. 1C).

### Associations between protein levels and ventricular volumes in healthy individuals

**Fig. 2:**
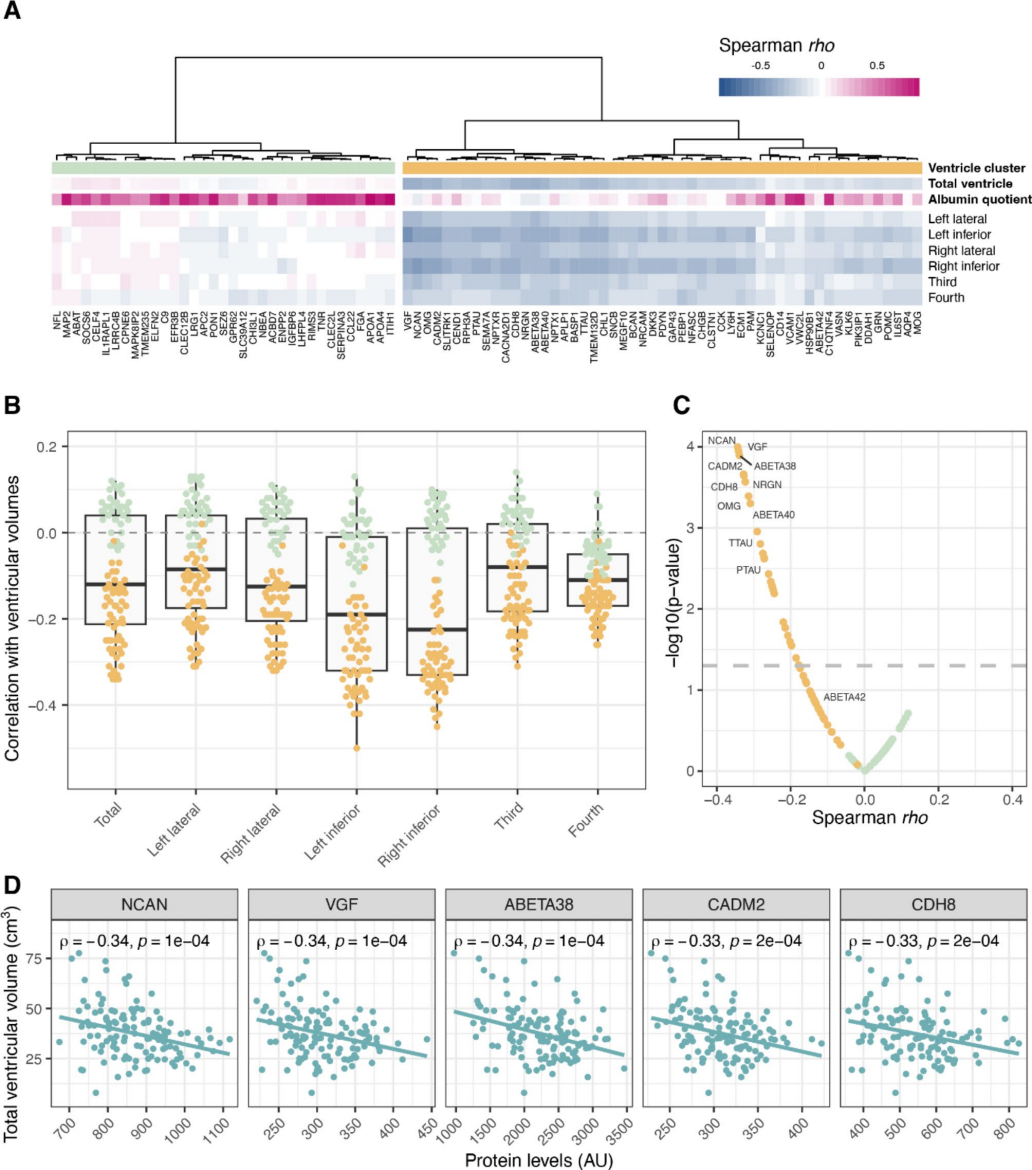
Association of ventricular volumes and CSF protein levels in A-T- individuals. **A**) A heatmap based on spearman correlation coefficients between protein levels and ventricular volumes. The heatmap is annotated with correlation per protein with total ventricular volume and correlation to albumin quotient. Two protein clusters were observed, one marked in green, and one marked in yellow. **B**) Correlation coefficients per protein and ventricular volume. **C**) Correlation coefficients and p-value for each protein against total ventricular volume. The horizontal dashed line indicates p-value = 0.05. **D**) Correlation between protein levels and total ventricular volume for the five proteins with strongest negative correlation

For initial investigation of associations between CSF proteins and ventricular volumes, levels of all 88 measured proteins were correlated with the separate ventricular volumes in A-T- individuals and the results summarised in a heatmap with protein clustering performed based on the correlation coefficients. The proteins were further annotated with correlation coefficients to total ventricular volume as well as albumin CSF/serum quotient (Fig. 2A). This analysis resulted in two main protein clusters, one including proteins with low correlation to ventricular volumes and high correlation to albumin quotient (green) and the other with negative correlation to ventricular volumes and low correlation to albumin quotient (yellow). Both clusters contained proteins with enriched expression in brain compared to other tissues. In addition, the green cluster included several proteins mainly produced in the liver, such as apolipoprotein A1 (APOA1) and inter-alpha-trypsin inhibitor heavy chain 1 (ITIH1) while the yellow cluster contained proteins known to be altered in AD, including the Aβ peptides, t-tau, p-tau, NRGN and neuromodulin/growth associated protein 43 (GAP43). The strongest associations were generally observed for the inferior horn of the lateral ventricles while the fourth ventricle showed the weakest correlation to proteins in this cluster (Fig. 2B). The protein with strongest negative correlation to total ventricular volume was neurocan (NCAN, rho = -0.34), followed by neurosecretory protein VGF (VGF, rho = -0.34), and Aβ38 (rho = -0.34) (Fig. 2C and D). Aβ40 also showed a significant negative correlation to total ventricular volume (rho = -0.31), as well as t-tau and p-tau (both rho = -0.28) while no significant correlation was observed for Aβ42 nor for NfL. See Supplementary Table 3 for correlation coefficients and p-values for all proteins. 2.

### Cortical thickness and white matter status and their association to protein levels

**Fig. 3:**
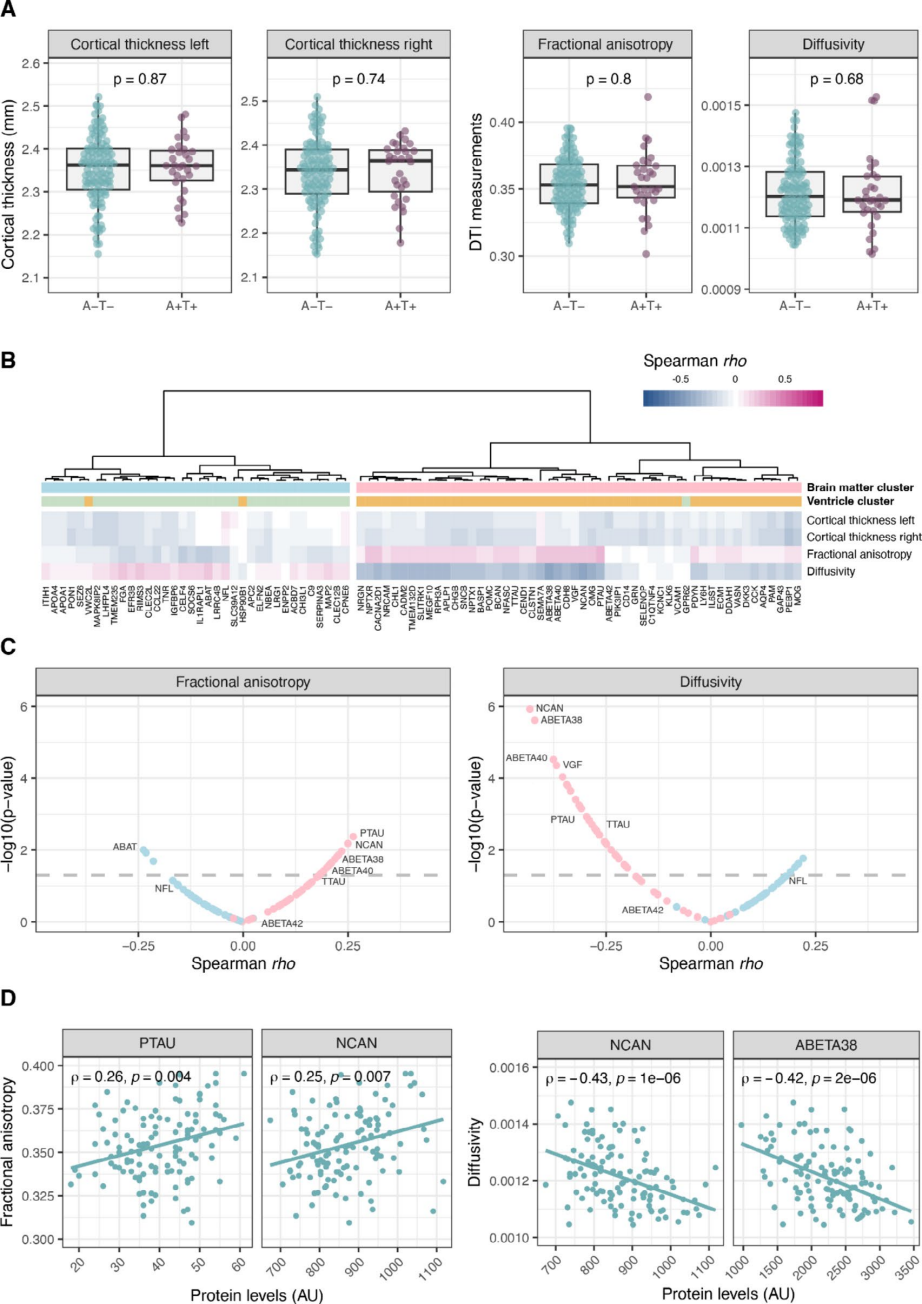
Association between CSF protein levels and grey and white matter status. **A**) No significant differences were observed for cortical thickness or white matter status measurements between the groups of A-T- and A + T + individuals. **B**) A heatmap based on spearman correlation coefficients between protein levels and cortical thickness and diffusion tensor imaging (DTI) measurements in healthy individuals. **C**) Correlation coefficients and p-value for each protein against fractional anisotropy and diffusivity. The horizontal dashed line indicates p-value = 0.05. **D**) Correlation between protein levels and diffusion tensor imaging measurements

We used the mean cortical thickness per hemisphere to assess the width of the grey matter in the cortex complemented by mean fractional anisotropy and mean diffusivity to assess the status of the white matter. The width of the grey matter in the two hemispheres showed a strong correlation in the A-T- group (rho = 0.96) but no significant correlations between the cortical thickness and total ventricular volume nor the DTI measurements were observed. The two measurements of water flow in white matter showed a strong negative correlation (rho = -0.74) but the correlation between mean diffusivity and total ventricular volume was even stronger (rho = 0.83). The correlation between total ventricular volume and mean fractional anisotropy was − 0.51 (Supplementary Fig. 3). No significant differences in grey or white matter status could be observed between the A-T- and A + T + individuals (Fig. 3A).

When correlating the measured protein levels in CSF with cortical thickness and DTI measurements, and again summarising the resulting correlation coefficients in a heatmap, two clusters were observed (Fig. 3B). The protein distribution between the clusters was largely similar to the results observed in the ventricular volume analysis. For the majority of proteins with negative correlation to ventricular volumes (yellow cluster in Fig. 2A) we observed negative correlation also to mean diffusivity and positive correlations to fractional anisotropy. Protein levels showed generally weak correlations to cortical thickness, ranging between − 0.25 and 0.11 where only phosphatidylethanolamine binding protein 1 (PEBP1) exhibited a significant correlation with both hemispheres (rho left = -0.21, rho right = -0.25). Cortical thickness did not seem to drive the clustering of the proteins (Fig. 3B). The strongest correlation between protein levels and fractional anisotropy was observed for p-tau (rho = 0.26), followed by NCAN (rho = 0.25), and a negative correlation with 4-aminobutyrate aminotransferase (ABAT) (rho = -0.24) (Fig. 3C and D). The protein with the strongest association to diffusivity was NCAN (rho = -0.43) followed by Aβ38 (rho = -0.42), Aβ40 (rho = -0.38) and VGF (rho = -0.37) (Fig. 3C and D). Neither NfL nor Aβ42 had a significant correlation to either fractional anisotropy or diffusivity. Supplementary Table 3 includes correlation with DTI measurements and p-values for all proteins.

### Associations in A-T- compared to A + T + individuals

**Fig. 4:**
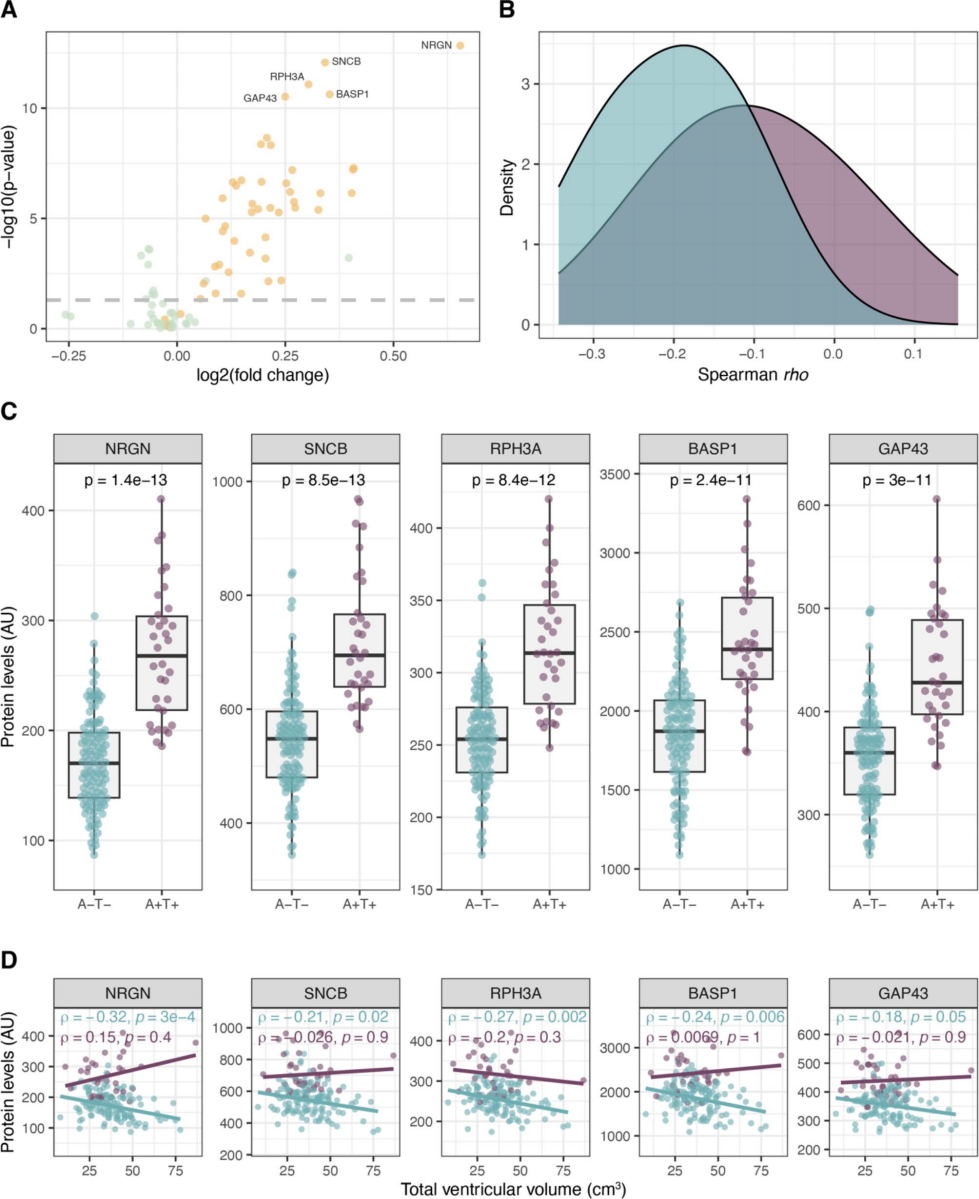
Comparisons between A-T- (turquoise) and A + T+ (purple). **A**) Proteins in the yellow ventricular volume associated cluster showed generally higher levels in A + T + individuals compared to the A-T- group. Yellow and green indicates cluster origin from the ventricular volume association analysis. The AD markers were excluded from this visualization. **B**) The distribution of correlation coefficients between protein levels and ventricular volume in the A-T- group and the A + T + group for the proteins in the ventricle associated (yellow) cluster. **C**) The five proteins with most significant differences in A + T + compared to A-T-. **D**) Protein correlations to total ventricular volume per sample group for the same five proteins

Finally, we aimed to investigate how the protein profiles and their associations with ventricular volume differed between A-T- and A + T + individuals. The majority of proteins in the ventricle associated (yellow) cluster showed significantly higher levels in the A + T + group compared to the A-T- group (Fig. 4A and Supplementary Table 4). Furthermore, we compared the correlation between levels of proteins from the ventricle associated cluster and total ventricular volume between the two sample groups and found that the correlations in the A + T + group were generally weaker and not statistically significant (Fig. 4B, Supplementary Table 5). The proteins with most significantly altered levels in the A + T + group were NRGN, synuclein beta (SNCB), rabphilin 3 A (RPH3A), brain abundant membrane attached signal protein 1 (BASP1) and GAP43 (Fig. 4C). All five proteins had a significant negative correlation to total ventricular volume among the A-T- individuals, ranging from rho of -0.32 to -0.18. However, this uniform trend was not observed in the A + T + group where all proteins showed comparably weaker correlations, with only one protein, RPH3A, showing negative correlation in the same range (rho = -0.2, Fig. 4D). Next, we assessed whether the differences in protein levels between the two groups could be enhanced if the putative non-disease source of protein variance was removed. Adjusting the protein levels based on total ventricular volume revealed p-values comparable to the unadjusted levels (data not shown).

## Discussion

This study aimed to investigate the potential influence of ventricular volumes on protein levels in CSF, in a cohort of cognitively healthy 70-year-olds with and without CSF AD pathology. Our findings revealed significant variations in ventricular volumes among the healthy individuals. Consistent with previous research, ventricular volumes positively correlated with ICV and were observed higher in males compared to females. To correct for head size, we adjusted the ventricular volumes by ICV using the residual approach which has been reported to be superior compared to the proportional approach wherein the volume of interest is divided with ICV [39, 40]. The correction for ICV reduced the observed sex-based differences.

Upon exploring the associations between CSF protein levels and ventricular volumes, two distinct protein clusters were observed. One cluster demonstrated negative correlations with ventricular volumes, suggesting a potential relationship between CSF protein levels and brain anatomy, possibly due to a dilution effect caused by larger ventricles. This pattern was not observed for proteins in the second cluster, in which proteins instead demonstrated a general positive correlation to albumin quotient. In a recent study aiming to predict brain to CSF protein secretion in healthy individuals, Waury and colleagues found proteins with functions related to adhesion, membrane and the extracellular space to more likely end up in CSF, including GAP43, NRGN and neuronal pentraxin 2 (NPTX2). On the contrary, NfL and other markers of neuronal injury were found less likely to be secreted [41]. While both our clusters included proteins expressed in the brain, NfL was found in one cluster and GAP43, NRGN and neuronal pentraxin 1 (NPTX1)/neuronal pentraxin receptor (NPTXR) in the other. One could thereby speculate that the different association patterns might reflect different causes of protein secretion or leakage. In line with this, 18 of the 33 proteins with elevated brain expression in the ventricle volume associated (yellow) cluster are annotated as actively secreted according to the Human Protein Atlas (www.proteinatlas.org, version 24) while only one in the other cluster. Other interesting aspects of protein origin in relation to potential dilution effects include regional expression patterns as well as cell-specificity. Future studies with larger and more unbiased panels of brain-derived proteins are needed to address these.

Our study unveiled significant negative correlations between ventricular volumes and amyloid and tau CSF markers, including t-tau, p-tau, Aβ38, and Aβ40. This finding contrasted with the results reported by Edsbagge et al. who observed significant negative correlations only for Aβ38 and Aβ40 but not for t-tau and p-tau, potentially due to their limited sample size (*n* = 19). Furthermore, van Waalwijk van Doorn et al. reported negative correlations between t-tau as well as p-tau and ventricular volumes within individuals diagnosed with AD and mild cognitive impairment, but not among healthy individuals. Although their group of healthy individuals was large, their utilization of the proportional ICV adjustment approach may have influenced the results.

In a recent study by Hansson et al., the correlations between lateral ventricular volumes and almost 400 CSF proteins were explored in a cohort of 720 individuals, including both cognitively healthy individuals and those with subjective or mild cognitive impairment. Using linear regression models adjusted for ICV, sex, age, and diagnostic group, they identified 64 proteins associated with ventricular volume. Comparing their results to ours, among the five proteins we observed with strongest correlation to total ventricular volume in the A-T- group (NCAN, VGF, Ab38, cell adhesion molecule 2 (CADM2) and cadherin 8 (CDH8)), lower levels of both VGF and Ab38 were also significantly associated with larger ventricular volumes in their study. Their study did however not identify NCAN as an associated protein, and neither CADM2 nor CDH8 were included in their analysis.

A decreased fractional anisotropy and an increased mean diffusivity indicates a reduced microstructural white matter integrity which is suggested to be associated with an increased neurological disease burden. Mean diffusivity displayed a strong correlation with total ventricular volume confirming an association between enlarged ventricular volume and increased diffusivity as previously observed by Coutu et al. [42]. Fractional anisotropy values close to one indicate diffusion predominantly along a single axis, whereas values close to zero indicate isotropic diffusion. Within our cohort, fractional anisotropy measurements ranged from 0.31 to 0.40, with a strong negative correlation observed with mean diffusivity. Altogether, these findings suggest that larger total ventricular volumes are associated with increased disruption of the white matter structural integrity. In line with this, the proteins with a negative correlation to ventricular volume, also correlated negatively to mean diffusivity and positively with fractional anisotropy. In contrast, our analysis revealed no correlation between cortical thickness and total ventricular volume and only weak correlations were observed between cortical thickness and CSF protein levels.

Further, we investigated whether any differences could be observed in protein profiles between A-T- and A + T + individuals and how protein levels were associated with ventricular volume in the A + T + group. We found that the majority of proteins included in the cluster associated with ventricular volume were increased in the A + T + group. As expected, since A + T + individuals did not show major cognitive impairment and represented pre-symptomatic AD patients, the two groups did not differ in ventricular volume measurements. The correlations between protein levels and CSF volumes were however different between the groups for the proteins most altered in the group comparison. When adjusting the protein levels for total ventricular volumes, we found no improvement in the discriminative performance of the proteins. This suggests that disease-related changes in CSF protein content might surpass the effect of CSF volume on protein levels. This remains to be further evaluated in a larger cohort also including patients with AD.

While this study provides valuable insights into the relationship between CSF protein profiles and ventricular volumes in cognitively healthy elderly and individuals with early AD pathology, several limitations should be acknowledged. Availability of longitudinal imaging trajectories could have strengthened the observed association with CSF protein levels and thus be more advantageous than the current cross-sectional imaging data. While the sample size for the A-T- is comparably large, the findings within the A + T + group may be hampered by the smaller sample size. The T + classification was in this study based on t-tau levels as the number of individuals with p-tau above the AD threshold were too few to perform statistical analysis. Furthermore, these findings are only based on a cohort of cognitively healthy individuals and future research efforts should focus on elucidating the association between CSF protein levels and ventricular volume in a cohort with diagnosed AD patients to cover the full spectrum of the disease. Nevertheless, ventricular volumes tend to increase with age which makes the studied cohort particularly suitable for this analysis, as all participants share the same age. However, the results may not be generalizable to other age groups.

In conclusion, this study sheds light on the intricate interplay between CSF protein profiles, brain anatomy, and early AD pathology. According to our findings, many brain-derived proteins are potentially subjected to ventricle volume-dependent dilution in CSF in cognitively healthy 70-year-olds, which may contribute to the inter-individual variation observed in studies of the CSF proteome.

## Electronic supplementary material

Below is the link to the electronic supplementary material.

Supplementary Material 1

## Data Availability

The datasets are not publicly available as they contain sensitive personal information. However, the datasets are available from the corresponding author on reasonable request for researchers who meet the criteria for access to sensitive personal data.

## Abbreviations

Ab: Amyloid beta
ABAT: 4-aminobutyrate aminotransferase
APOA1: Apolipoprotein A1
BASP1: Brain abundant membrane attached signal protein 1
CADM2: Cell adhesion molecule 2
CDH8: Cadherin 8
CDR: Clinical Dementia Rating
CSF: Cerebrospinal fluid
DTI: Diffusion tensor imaging
GAP43: Neuromodulin/growth associated protein 43
ICV: Intracranial volume
iNPH: Idiopathic normal pressure hydrocephalus
ITIH1: Inter-alpha-trypsin inhibitor heavy chain 1
MCI: Mild cognitive impairment
MMSE: Mini-Mental State Examination
MRI: Magnetic resonance imaging
NfL: Neurofilament light chain
NPTX1: Neuronal pentraxin 1
NPTX2: Neuronal pentraxin 2
NPTXR: Neuronal pentraxin receptor
NRGN: Neurogranin
PEBP1: Phosphatidylethanolamine binding protein 1
p-tau: Phosphorylated tau
RPH3A: Rabphilin 3 A
SNCB: Synuclein beta
t-tau: Total tau
VGF: Neurosecretory protein VGF
