# Supplementary material for: CSF levels of brain-derived proteins correlate with brain ventricular volume in cognitively healthy 70-year-olds
Source: Clin Proteomics. 2024 Dec 12;21:65. doi: 10.1186/s12014-024-09517-1 (PMC11636040; doi:10.1186/s12014-024-09517-1)
Supplement: Supplementary file 1 — Supplementary Material 1 [file 12014_2024_9517_MOESM1_ESM.docx]

# Supplementary figures


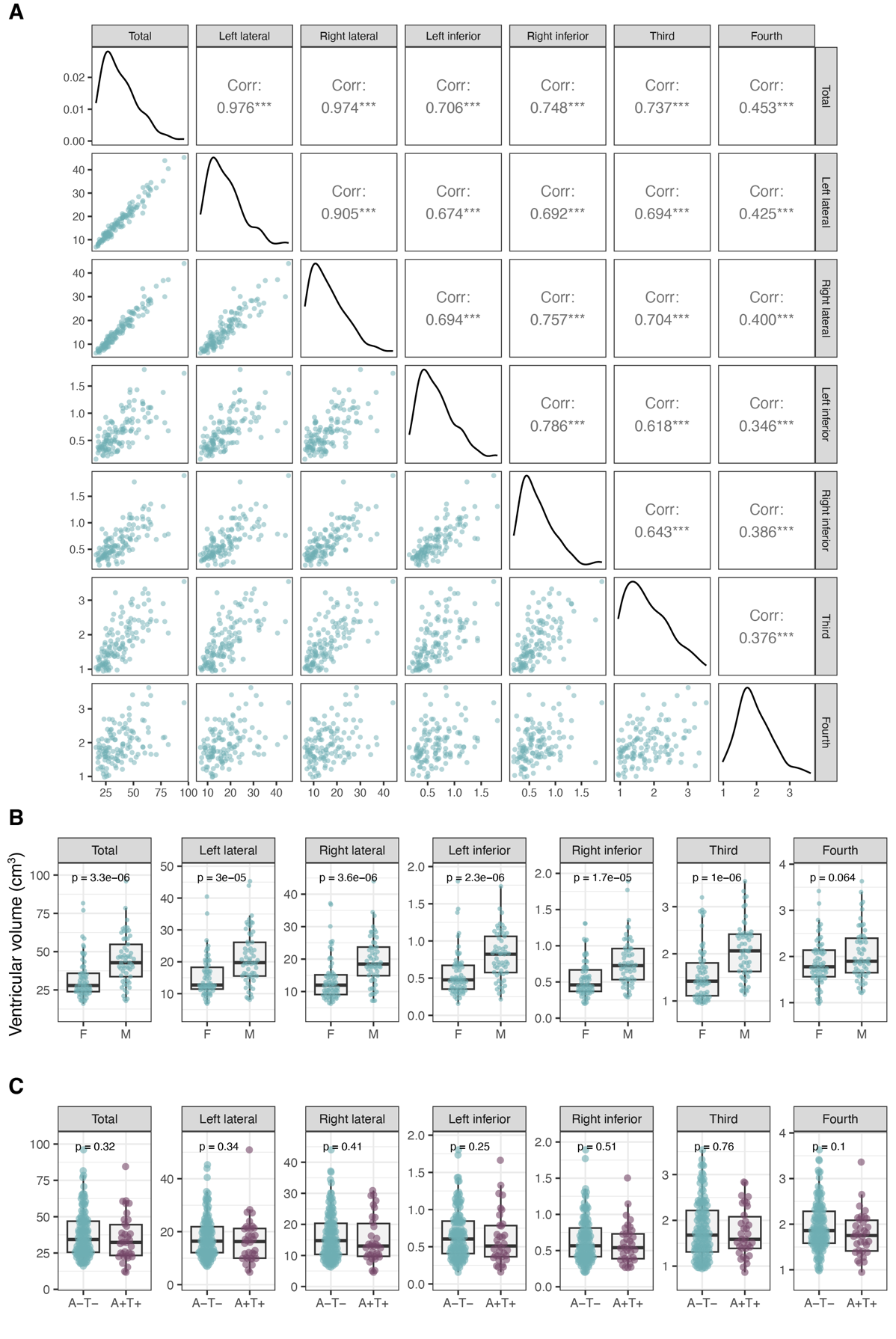


**Supplementary figure 1:** *A) Correlation between ventricular volumes among healthy individuals. B) Larger ventricular volumes were observed in males. C) No significant differences in ventricular volumes between the two sample groups.*


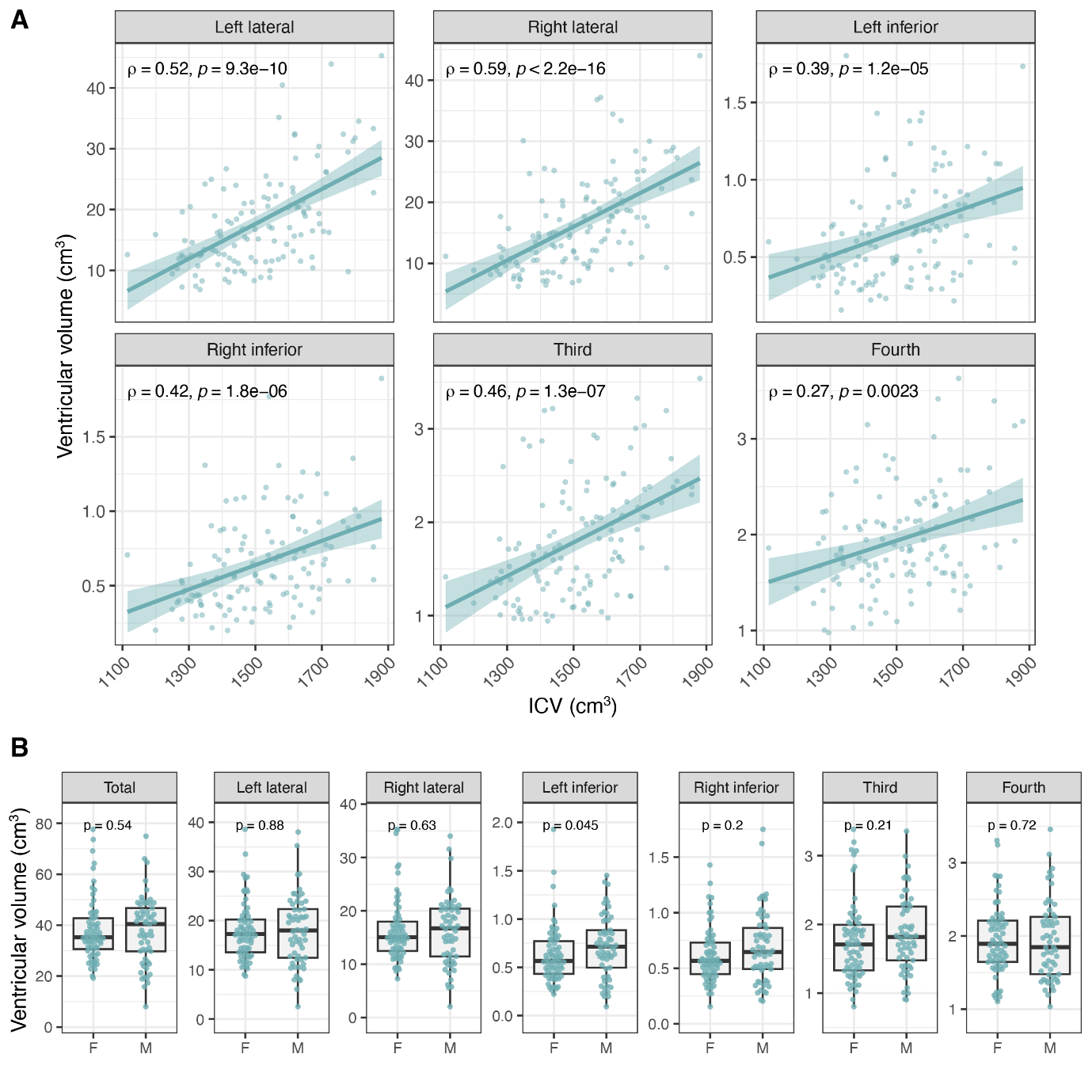


**Supplementary figure 2**: *Ventricular volumes in relation to ICV. A) Correlation between ventricular volumes and intracranial volumes (ICV) in healthy individuals. B) No sex differences were observed for ventricular volumes after ICV adjustment.*


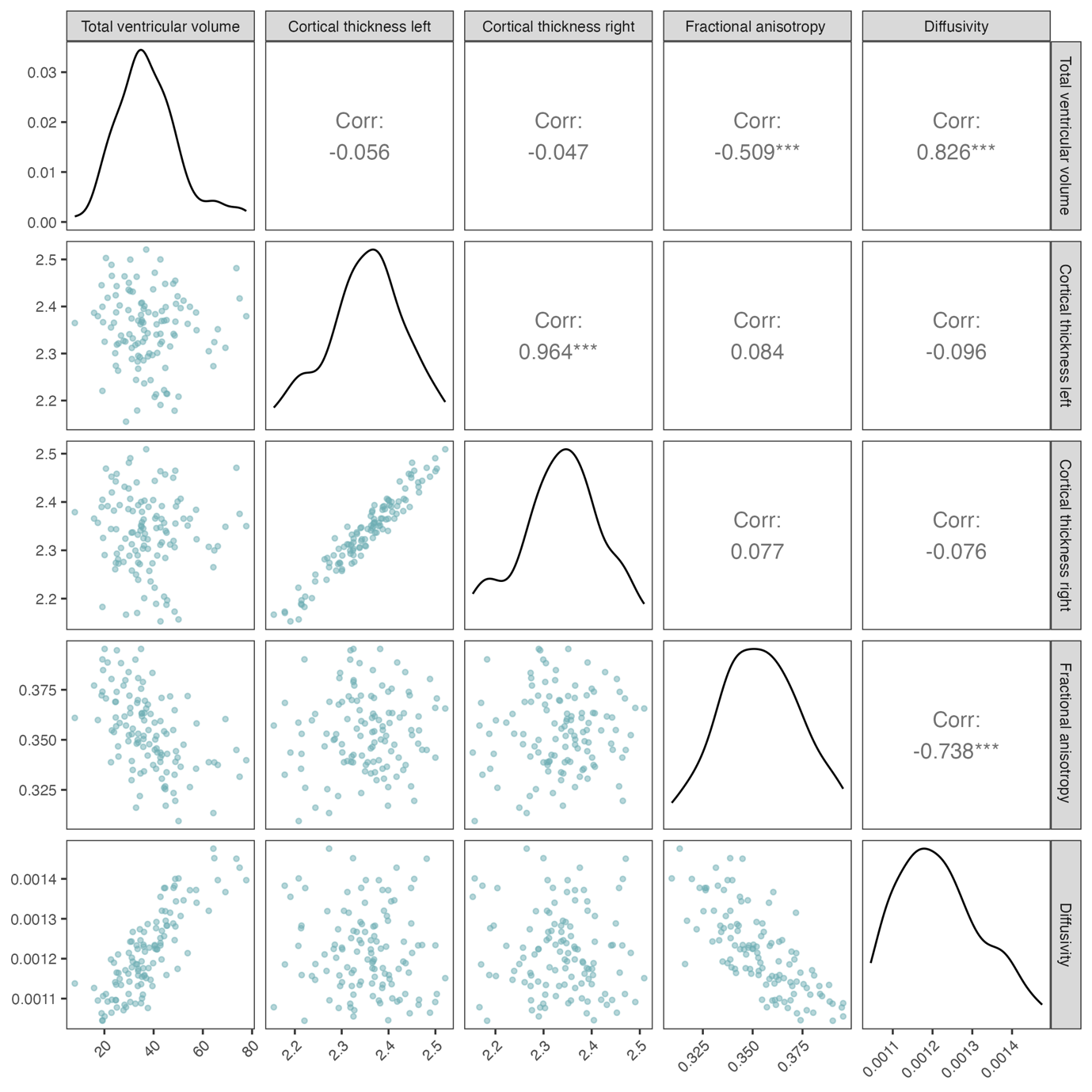


**Supplementary figure 3**: *Correlation between total ventricular volume, cortical thickness (left and right hemisphere), and white matter status measured by diffuse tensor imaging.*

# Supplementary tables

**Supplementary table 1**: *Proteins measured with suspension bead array*

**Protein Antibody Protein long name Uniprot ID**

ABAT HPA041528 4-aminobutyrate aminotransferase P80404

ACBD7 HPA062478 Acyl-CoA binding domain containing 7 Q8N6N7

APC2 HPA050820 APC regulator of WNT signaling pathway 2 O95996

APLP1 HPA028971 Amyloid beta precursor like protein 1 P51693

APOA1 HPA046715 Apolipoprotein A1 P02647

APOA4 HPA005149 Apolipoprotein A4 P06727

AQP4 HPA014784 Aquaporin 4 P55087

BASP1 HPA050333 Brain abundant membrane attached signal protein 1 P80723

BCAN HPA007865 Brevican Q96GW7

C1QTNF4 HPA041032 C1q and TNF related 4 Q9BXJ3

C9 HPA029577 Complement C9 P02748

CACNA2D1 HPA008213 Calcium voltage-gated channel auxiliary subunit alpha2delta 1 P54289

CADM2 HPA010024 Cell adhesion molecule 2 Q8N3J6

CCK HPA069515 Cholecystokinin P06307

CCL22 HPA077819 C-C motif chemokine ligand 22 O00626

CD14 HPA002035 CD14 molecule P08571

CDH8 HPA014908 Cadherin 8 P55286

CELF4 HPA037986 CUGBP Elav-like family member 4 Q9BZC1

CEND1 HPA042527 Cell cycle exit and neuronal differentiation 1 Q8N111

CHGB HPA012872 Chromogranin B P05060

CHI3L1 HPA072269 Chitinase 3 like 1 P36222

CHL1 HPA003345 Cell adhesion molecule L1 like O00533

CLEC12B HPA026973 C-type lectin domain family 12 member B Q2HXU8

CLEC2L HPA045050 C-type lectin domain family 2 member L P0C7M8

CLSTN1 HPA012749 Calsyntenin 1 O94985

CPNE6 HPA031636 Copine 6 O95741

DDAH1 HPA071064 Dimethylarginine dimethylaminohydrolase 1 O94760

DKK3 HPA011164 Dickkopf WNT signaling pathway inhibitor 3 Q9UBP4

ECM1 HPA027241 Extracellular matrix protein 1 Q16610

EFR3B HPA038089 EFR3 homolog B Q9Y2G0

ELFN2 HPA000781 Extracellular leucine rich repeat and fibronectin type III domain containing 2 Q5R3F8

ENPP2 HPA053652 Ectonucleotide pyrophosphatase/phosphodiesterase 2 Q13822

FGA HPA051370 Fibrinogen alpha chain P02671

GAP43 PA5-34943 Neuromodulin/growth associated protein 43 P17677

GPR62 HPA031309 G protein-coupled receptor 62 Q9BZJ7

GRN HPA008763 Granulin precursor P28799

HSP90B1 HPA003901 Heat shock protein 90 beta family member 1 P14625

IGFBP6 HPA075088 Insulin like growth factor binding protein 6 P24592

IL1RAPL1 HPA000564 Interleukin 1 receptor accessory protein like 1 Q9NZN1

IL6ST HPA010030 Interleukin 6 signal transducer P40189

ITIH1 HPA042049 Inter-alpha-trypsin inhibitor heavy chain 1 P19827

KCNC1 HPA041392 Potassium voltage-gated channel subfamily C member 1 P48547

KLK6 HPA019525 Kallikrein related peptidase 6 Q92876

LHFPL4 HPA041421 LHFPL tetraspan subfamily member 4 Q7Z7J7

LRG1 HPA001888 Leucine rich alpha-2-glycoprotein 1 P02750

LRRC4B HPA058986 Leucine rich repeat containing 4B Q9NT99

LY6H HPA077218 Lymphocyte antigen 6 family member H O94772

MAP2 HPA012558 Microtubule associated protein 2 P11137

MAPK8IP2 HPA003572 Mitogen-activated protein kinase 8 interacting protein 2 Q13387

MEGF10 HPA026876 Multiple EGF like domains 10 Q96KG7

MOG AMAb91067 Myelin oligodendrocyte glycoprotein Q16653

NBEA HPA040385 Neurobeachin Q8NFP9

NCAN HPA058000 Neurocan O14594

NFASC HPA073444 Neurofascin O94856

NPTX1 HPA077062 Neuronal pentraxin 1 Q15818

NPTXR HPA001079 Neuronal pentraxin receptor O95502

NRCAM HPA061433 Neuronal cell adhesion molecule Q92823

OMG HPA008206 Oligodendrocyte myelin glycoprotein P23515

PAM HPA042260 Peptidylglycine alpha-amidating monooxygenase P19021

PDYN HPA053342 Prodynorphin P01213

PEBP1 HPA063904 Phosphatidylethanolamine binding protein 1 P30086

PIK3IP1 HPA002959 Phosphoinositide-3-kinase interacting protein 1 Q96FE7

POMC HPA063644 Proopiomelanocortin P01189

PON1 HPA001640 Paraoxonase 1 P27169

RIMS3 HPA055285 Regulating synaptic membrane exocytosis 3 Q9UJD0

RPH3A HPA002475 Rabphilin 3A Q9Y2J0

SELENOP HPA058160 Selenoprotein P P49908

SEMA7A HPA042273 Semaphorin 7A (John Milton Hagen blood group) O75326

SERPINA3 HPA000893 Serpin family A member 3 P01011

SEZ6 HPA007703 Seizure related 6 homolog Q53EL9

SLC39A12 HPA059726 Solute carrier family 39 member 12 Q504Y0

SLITRK1 HPA012414 SLIT and NTRK like family member 1 Q96PX8

SNCB HPA035876 Synuclein beta Q16143

SOCS6 HPA035477 Suppressor of cytokine signaling 6 O14544

TMEM132D HPA010739 Transmembrane protein 132D Q14C87

TMEM235 HPA053939 Transmembrane protein 235 A6NFC5

TNR HPA027150 Tenascin R Q92752

VASN HPA011246 Vasorin Q6EMK4

VCAM1 HPA069867 Vascular cell adhesion molecule 1 P19320

VGF HPA055177 Neurosecretory protein VGF O15240

VWC2L HPA059414 Von Willebrand factor C domain containing 2 like B2RUY7

**Supplementary table 2**: *Measurements of ventricular volumes in healthy individuals based on raw volume measurements.*

| **Ventricular volume** | **ml, median [range]** |
| --- | --- |
| Total | 34.34 [16.02-96.00] |
| Left lateral | 16.42 [6.86-45.28] |
| Left inferior | 0.60 [0.16-1.80] |
| Right lateral | 14.78 [6.26-44.00] |
| Right inferior | 0.57 [0.20-1.89] |
| Third | 1.68 [0.94-3.54] |
| Fourth | 1.86 [0.98-3.63] |

**Supplementary table 3**: *Summary statistics used for volcano plots in Figure 2C and Figure 3C.*

|  | **Total ventricular volume** | | **Fractional anisotropy** | | **Diffusivity** | |
| --- | --- | --- | --- | --- | --- | --- |
| **Protein** | **Spearman *rho*** | **p-value** | **Spearman *rho*** | **p-value** | **Spearman *rho*** | **p-value** |
| ABAT | 0.11 | 2E-01 | -0.24 | 1E-02 | 0.21 | 2E-02 |
| ABETA38 | -0.34 | 1E-04 | 0.22 | 2E-02 | -0.42 | 2E-06 |
| ABETA40 | -0.31 | 5E-04 | 0.21 | 3E-02 | -0.38 | 3E-05 |
| ABETA42 | -0.12 | 2E-01 | 0.02 | 9E-01 | -0.13 | 2E-01 |
| ACBD7 | 0.02 | 9E-01 | -0.06 | 5E-01 | 0.11 | 3E-01 |
| APC2 | -0.02 | 9E-01 | -0.09 | 3E-01 | 0.04 | 7E-01 |
| APLP1 | -0.25 | 5E-03 | 0.18 | 5E-02 | -0.31 | 6E-04 |
| APOA1 | 0.03 | 7E-01 | -0.01 | 9E-01 | 0.10 | 3E-01 |
| APOA4 | 0.05 | 6E-01 | 0.00 | 1E+00 | 0.09 | 3E-01 |
| AQP4 | -0.14 | 1E-01 | 0.06 | 5E-01 | -0.18 | 5E-02 |
| BASP1 | -0.24 | 6E-03 | 0.13 | 2E-01 | -0.25 | 6E-03 |
| BCAN | -0.22 | 1E-02 | 0.19 | 4E-02 | -0.22 | 1E-02 |
| C1QTNF4 | -0.10 | 3E-01 | -0.02 | 8E-01 | 0.01 | 9E-01 |
| C9 | 0.08 | 4E-01 | -0.11 | 2E-01 | 0.15 | 1E-01 |
| CACNA2D1 | -0.27 | 2E-03 | 0.21 | 2E-02 | -0.34 | 2E-04 |
| CADM2 | -0.33 | 2E-04 | 0.16 | 8E-02 | -0.31 | 7E-04 |
| CCK | -0.17 | 7E-02 | 0.07 | 4E-01 | -0.21 | 3E-02 |
| CCL22 | 0.06 | 5E-01 | -0.15 | 1E-01 | 0.18 | 6E-02 |
| CD14 | -0.07 | 4E-01 | 0.00 | 1E+00 | -0.05 | 6E-01 |
| CDH8 | -0.33 | 2E-04 | 0.22 | 2E-02 | -0.34 | 1E-04 |
| CELF4 | 0.10 | 3E-01 | -0.23 | 1E-02 | 0.17 | 6E-02 |
| CEND1 | -0.25 | 5E-03 | 0.15 | 1E-01 | -0.23 | 1E-02 |
| CHGB | -0.16 | 8E-02 | 0.19 | 5E-02 | -0.25 | 6E-03 |
| CHI3L1 | -0.03 | 8E-01 | -0.07 | 5E-01 | 0.09 | 4E-01 |
| CHL1 | -0.27 | 2E-03 | 0.16 | 8E-02 | -0.30 | 1E-03 |
| CLEC12B | 0.00 | 1E+00 | -0.06 | 5E-01 | 0.12 | 2E-01 |
| CLEC2L | 0.05 | 6E-01 | -0.11 | 2E-01 | 0.18 | 5E-02 |
| CLSTN1 | -0.17 | 5E-02 | 0.14 | 1E-01 | -0.25 | 7E-03 |
| CPNE6 | 0.04 | 7E-01 | -0.09 | 3E-01 | 0.13 | 2E-01 |
| DDAH1 | -0.14 | 1E-01 | 0.11 | 3E-01 | -0.18 | 6E-02 |
| DKK3 | -0.17 | 5E-02 | 0.10 | 3E-01 | -0.17 | 6E-02 |
| ECM1 | -0.14 | 1E-01 | 0.11 | 2E-01 | -0.14 | 1E-01 |
| EFR3B | 0.07 | 4E-01 | -0.17 | 7E-02 | 0.19 | 4E-02 |
| ELFN2 | 0.07 | 4E-01 | -0.12 | 2E-01 | 0.10 | 3E-01 |
| ENPP2 | 0.03 | 7E-01 | -0.04 | 6E-01 | 0.05 | 6E-01 |
| FGA | 0.10 | 3E-01 | -0.16 | 9E-02 | 0.21 | 2E-02 |
| GAP43 | -0.18 | 5E-02 | 0.10 | 3E-01 | -0.17 | 6E-02 |
| GPR62 | -0.03 | 7E-01 | -0.02 | 8E-01 | 0.02 | 8E-01 |
| GRN | -0.15 | 1E-01 | 0.01 | 9E-01 | -0.07 | 5E-01 |
| HSP90B1 | -0.13 | 2E-01 | 0.02 | 8E-01 | -0.08 | 4E-01 |
| IGFBP6 | 0.07 | 4E-01 | -0.14 | 1E-01 | 0.12 | 2E-01 |
| IL1RAPL1 | 0.09 | 3E-01 | -0.21 | 2E-02 | 0.18 | 5E-02 |
| IL6ST | -0.13 | 1E-01 | 0.08 | 4E-01 | -0.13 | 1E-01 |
| ITIH1 | 0.06 | 5E-01 | -0.03 | 7E-01 | 0.13 | 2E-01 |
| KCNC1 | -0.11 | 2E-01 | -0.02 | 9E-01 | 0.00 | 1E+00 |
| KLK6 | -0.09 | 3E-01 | 0.02 | 8E-01 | -0.03 | 7E-01 |
| LHFPL4 | 0.03 | 8E-01 | -0.08 | 4E-01 | 0.12 | 2E-01 |
| LRG1 | 0.03 | 8E-01 | -0.03 | 7E-01 | 0.06 | 5E-01 |
| LRRC4B | 0.11 | 2E-01 | -0.16 | 9E-02 | 0.15 | 1E-01 |
| LY6H | -0.17 | 7E-02 | 0.07 | 4E-01 | -0.20 | 3E-02 |
| MAP2 | 0.06 | 5E-01 | -0.11 | 2E-01 | 0.16 | 9E-02 |
| MAPK8IP2 | 0.06 | 5E-01 | -0.07 | 4E-01 | 0.13 | 2E-01 |
| MEGF10 | -0.21 | 2E-02 | 0.20 | 3E-02 | -0.30 | 1E-03 |
| MOG | -0.11 | 2E-01 | 0.13 | 2E-01 | -0.17 | 7E-02 |
| NBEA | 0.04 | 6E-01 | -0.11 | 3E-01 | 0.06 | 5E-01 |
| NCAN | -0.34 | 1E-04 | 0.25 | 7E-03 | -0.43 | 1E-06 |
| NFASC | -0.21 | 2E-02 | 0.16 | 9E-02 | -0.27 | 3E-03 |
| NFL | 0.05 | 6E-01 | -0.16 | 9E-02 | 0.17 | 7E-02 |
| NPTX1 | -0.25 | 5E-03 | 0.18 | 5E-02 | -0.28 | 3E-03 |
| NPTXR | -0.25 | 5E-03 | 0.21 | 2E-02 | -0.29 | 1E-03 |
| NRCAM | -0.20 | 3E-02 | 0.15 | 1E-01 | -0.28 | 2E-03 |
| NRGN | -0.32 | 3E-04 | 0.09 | 3E-01 | -0.29 | 2E-03 |
| OMG | -0.31 | 4E-04 | 0.23 | 1E-02 | -0.35 | 9E-05 |
| PAM | -0.16 | 8E-02 | 0.10 | 3E-01 | -0.20 | 3E-02 |
| PDYN | -0.20 | 3E-02 | 0.20 | 3E-02 | -0.20 | 3E-02 |
| PEBP1 | -0.19 | 4E-02 | 0.14 | 1E-01 | -0.24 | 1E-02 |
| PIK3IP1 | -0.12 | 2E-01 | 0.02 | 8E-01 | -0.10 | 3E-01 |
| POMC | -0.13 | 1E-01 | 0.15 | 1E-01 | -0.22 | 2E-02 |
| PON1 | 0.00 | 1E+00 | -0.01 | 9E-01 | 0.10 | 3E-01 |
| PTAU | -0.28 | 2E-03 | 0.26 | 4E-03 | -0.31 | 6E-04 |
| RIMS3 | 0.05 | 6E-01 | -0.12 | 2E-01 | 0.22 | 2E-02 |
| RPH3A | -0.27 | 2E-03 | 0.19 | 4E-02 | -0.32 | 4E-04 |
| SELENOP | -0.02 | 8E-01 | 0.02 | 8E-01 | 0.02 | 8E-01 |
| SEMA7A | -0.25 | 6E-03 | 0.24 | 1E-02 | -0.31 | 6E-04 |
| SERPINA3 | 0.06 | 5E-01 | -0.11 | 2E-01 | 0.13 | 2E-01 |
| SEZ6 | -0.04 | 6E-01 | -0.07 | 5E-01 | 0.09 | 3E-01 |
| SLC39A12 | -0.03 | 7E-01 | -0.02 | 9E-01 | -0.01 | 9E-01 |
| SLITRK1 | -0.26 | 4E-03 | 0.18 | 5E-02 | -0.31 | 7E-04 |
| SNCB | -0.21 | 2E-02 | 0.18 | 5E-02 | -0.27 | 3E-03 |
| SOCS6 | 0.12 | 2E-01 | -0.17 | 7E-02 | 0.20 | 3E-02 |
| TMEM132D | -0.29 | 1E-03 | 0.18 | 5E-02 | -0.33 | 2E-04 |
| TMEM235 | 0.07 | 5E-01 | -0.13 | 2E-01 | 0.17 | 7E-02 |
| TNR | 0.05 | 6E-01 | -0.09 | 3E-01 | 0.17 | 7E-02 |
| TTAU | -0.28 | 2E-03 | 0.18 | 6E-02 | -0.27 | 4E-03 |
| VASN | -0.13 | 1E-01 | 0.11 | 2E-01 | -0.17 | 7E-02 |
| VCAM1 | -0.09 | 3E-01 | -0.02 | 8E-01 | 0.04 | 6E-01 |
| VGF | -0.34 | 1E-04 | 0.21 | 2E-02 | -0.37 | 4E-05 |
| VWC2L | -0.07 | 5E-01 | -0.07 | 5E-01 | 0.08 | 4E-01 |

**Supplementary table 4**: *Summary statistics used for volcano plots in Figure 4A. Values corresponding to a non-significant p-value (0.05) marked with grey. Cluster 1 corresponds to the ventricular volume associated cluster.*

| **Protein** | **p-value** | **-log10(p-value)** | **Fold change** | **Log2(Fold change)** | **Cluster** |
| --- | --- | --- | --- | --- | --- |
| ABAT | 6E-01 | 0.22 | 0.98 | -0.03 | 2 |
| ACBD7 | 6E-01 | 0.20 | 0.99 | -0.01 | 2 |
| APC2 | 2E-01 | 0.71 | 0.99 | -0.02 | 2 |
| APLP1 | 1E-05 | 5.00 | 1.05 | 0.07 | 1 |
| APOA1 | 9E-01 | 0.04 | 0.97 | -0.04 | 2 |
| APOA4 | 3E-01 | 0.54 | 1.00 | 0.00 | 2 |
| AQP4 | 5E-06 | 5.27 | 1.18 | 0.23 | 1 |
| BASP1 | 2E-11 | 10.63 | 1.28 | 0.35 | 1 |
| BCAN | 3E-03 | 2.55 | 1.09 | 0.12 | 1 |
| C1QTNF4 | 2E-01 | 0.66 | 1.01 | 0.01 | 1 |
| C9 | 6E-01 | 0.20 | 1.02 | 0.02 | 2 |
| CACNA2D1 | 3E-06 | 5.47 | 1.16 | 0.22 | 1 |
| CADM2 | 2E-07 | 6.66 | 1.14 | 0.20 | 1 |
| CCK | 7E-07 | 6.14 | 1.32 | 0.40 | 1 |
| CCL22 | 3E-02 | 1.54 | 0.96 | -0.05 | 2 |
| CD14 | 7E-03 | 2.14 | 1.16 | 0.21 | 1 |
| CDH8 | 6E-08 | 7.19 | 1.20 | 0.27 | 1 |
| CELF4 | 3E-01 | 0.55 | 0.84 | -0.25 | 2 |
| CEND1 | 4E-09 | 8.36 | 1.14 | 0.19 | 1 |
| CHGB | 2E-03 | 2.81 | 1.06 | 0.09 | 1 |
| CHI3L1 | 7E-02 | 1.13 | 0.98 | -0.03 | 2 |
| CHL1 | 6E-08 | 7.21 | 1.33 | 0.41 | 1 |
| CLEC12B | 5E-01 | 0.29 | 1.03 | 0.04 | 2 |
| CLEC2L | 5E-02 | 1.34 | 0.95 | -0.07 | 2 |
| CLSTN1 | 2E-06 | 5.75 | 1.21 | 0.27 | 1 |
| CPNE6 | 2E-02 | 1.72 | 0.96 | -0.05 | 2 |
| DDAH1 | 1E-06 | 5.91 | 1.08 | 0.11 | 1 |
| DKK3 | 2E-07 | 6.64 | 1.09 | 0.13 | 1 |
| ECM1 | 5E-06 | 5.28 | 1.13 | 0.17 | 1 |
| EFR3B | 1E-03 | 2.90 | 0.95 | -0.07 | 2 |
| ELFN2 | 6E-01 | 0.25 | 0.97 | -0.05 | 2 |
| ENPP2 | 9E-01 | 0.04 | 0.99 | -0.01 | 2 |
| FGA | 6E-01 | 0.24 | 0.97 | -0.04 | 2 |
| GAP43 | 3E-11 | 10.52 | 1.19 | 0.25 | 1 |
| GPR62 | 2E-01 | 0.71 | 0.99 | -0.01 | 2 |
| GRN | 1E-03 | 2.90 | 1.07 | 0.10 | 1 |
| HSP90B1 | 7E-03 | 2.18 | 1.18 | 0.24 | 1 |
| IGFBP6 | 3E-01 | 0.51 | 1.02 | 0.03 | 2 |
| IL1RAPL1 | 4E-01 | 0.45 | 0.96 | -0.05 | 2 |
| IL6ST | 4E-05 | 4.41 | 1.08 | 0.11 | 1 |
| ITIH1 | 6E-01 | 0.22 | 0.93 | -0.11 | 2 |
| KCNC1 | 4E-01 | 0.41 | 0.98 | -0.03 | 1 |
| KLK6 | 9E-03 | 2.05 | 1.04 | 0.06 | 1 |
| LHFPL4 | 3E-04 | 3.58 | 0.96 | -0.06 | 2 |
| LRG1 | 7E-01 | 0.17 | 0.95 | -0.07 | 2 |
| LRRC4B | 2E-01 | 0.63 | 0.84 | -0.26 | 2 |
| LY6H | 5E-09 | 8.32 | 1.16 | 0.22 | 1 |
| MAP2 | 9E-01 | 0.06 | 0.99 | -0.01 | 2 |
| MAPK8IP2 | 2E-04 | 3.61 | 0.96 | -0.07 | 2 |
| MEGF10 | 7E-05 | 4.14 | 1.15 | 0.20 | 1 |
| MOG | 3E-02 | 1.58 | 1.11 | 0.15 | 1 |
| NBEA | 6E-01 | 0.24 | 0.99 | -0.01 | 2 |
| NCAN | 3E-07 | 6.48 | 1.10 | 0.14 | 1 |
| NFASC | 3E-07 | 6.59 | 1.19 | 0.25 | 1 |
| NFL | 6E-04 | 3.21 | 1.32 | 0.40 | 2 |
| NPTX1 | 1E-04 | 3.97 | 1.10 | 0.13 | 1 |
| NPTXR | 2E-06 | 5.66 | 1.13 | 0.17 | 1 |
| NRCAM | 4E-06 | 5.38 | 1.25 | 0.33 | 1 |
| NRGN | 1E-13 | 12.84 | 1.57 | 0.65 | 1 |
| OMG | 7E-07 | 6.14 | 1.26 | 0.33 | 1 |
| PAM | 4E-06 | 5.42 | 1.14 | 0.19 | 1 |
| PDYN | 7E-04 | 3.18 | 1.15 | 0.20 | 1 |
| PEBP1 | 2E-07 | 6.72 | 1.11 | 0.15 | 1 |
| PIK3IP1 | 2E-05 | 4.65 | 1.08 | 0.11 | 1 |
| POMC | 3E-02 | 1.60 | 1.06 | 0.09 | 1 |
| PON1 | 7E-01 | 0.13 | 0.98 | -0.03 | 2 |
| RIMS3 | 2E-01 | 0.67 | 0.96 | -0.06 | 2 |
| RPH3A | 8E-12 | 11.08 | 1.23 | 0.30 | 1 |
| SELENOP | 4E-02 | 1.35 | 1.04 | 0.05 | 1 |
| SEMA7A | 4E-04 | 3.45 | 1.12 | 0.17 | 1 |
| SERPINA3 | 6E-01 | 0.24 | 1.01 | 0.02 | 2 |
| SEZ6 | 6E-01 | 0.19 | 0.99 | -0.02 | 2 |
| SLC39A12 | 7E-03 | 2.16 | 1.05 | 0.07 | 2 |
| SLITRK1 | 2E-09 | 8.66 | 1.15 | 0.21 | 1 |
| SNCB | 8E-13 | 12.07 | 1.27 | 0.34 | 1 |
| SOCS6 | 3E-02 | 1.57 | 0.96 | -0.06 | 2 |
| TMEM132D | 5E-08 | 7.29 | 1.33 | 0.41 | 1 |
| TMEM235 | 5E-04 | 3.31 | 0.94 | -0.08 | 2 |
| TNR | 9E-02 | 1.06 | 0.96 | -0.06 | 2 |
| VASN | 3E-06 | 5.48 | 1.21 | 0.27 | 1 |
| VCAM1 | 7E-01 | 0.14 | 0.98 | -0.02 | 1 |
| VGF | 6E-07 | 6.20 | 1.20 | 0.26 | 1 |
| VWC2L | 9E-01 | 0.07 | 0.99 | -0.02 | 1 |

**Supplementary table 5**: *Correlation between total ventricular volume and protein levels per sample group for the proteins in the ventricular volume associated cluster. Values corresponding to a non-significant p-value (0.05) marked with grey.*

|  | **A-T-** | | **A+T+** | |
| --- | --- | --- | --- | --- |
| **Protein** | **Spearman *rho*** | **p-value** | **Spearman *rho*** | **p-value** |
| ABETA38 | -0.34 | 1E-04 | 0.04 | 8E-01 |
| ABETA40 | -0.31 | 5E-04 | -0.02 | 9E-01 |
| ABETA42 | -0.12 | 2E-01 | -0.03 | 9E-01 |
| APLP1 | -0.25 | 5E-03 | -0.13 | 5E-01 |
| AQP4 | -0.14 | 1E-01 | -0.11 | 5E-01 |
| BASP1 | -0.24 | 6E-03 | 0.01 | 1E+00 |
| BCAN | -0.22 | 1E-02 | -0.17 | 3E-01 |
| C1QTNF4 | -0.10 | 3E-01 | -0.16 | 4E-01 |
| CACNA2D1 | -0.27 | 2E-03 | -0.19 | 3E-01 |
| CADM2 | -0.33 | 2E-04 | -0.14 | 4E-01 |
| CCK | -0.17 | 7E-02 | -0.06 | 7E-01 |
| CD14 | -0.07 | 4E-01 | -0.22 | 2E-01 |
| CDH8 | -0.33 | 2E-04 | -0.09 | 6E-01 |
| CEND1 | -0.25 | 5E-03 | -0.01 | 9E-01 |
| CHGB | -0.16 | 8E-02 | -0.13 | 4E-01 |
| CHL1 | -0.27 | 2E-03 | -0.23 | 2E-01 |
| CLSTN1 | -0.17 | 5E-02 | -0.28 | 1E-01 |
| DDAH1 | -0.14 | 1E-01 | -0.10 | 6E-01 |
| DKK3 | -0.17 | 5E-02 | -0.21 | 2E-01 |
| ECM1 | -0.14 | 1E-01 | -0.11 | 5E-01 |
| GAP43 | -0.18 | 5E-02 | -0.02 | 9E-01 |
| GRN | -0.15 | 1E-01 | -0.13 | 5E-01 |
| HSP90B1 | -0.13 | 2E-01 | -0.19 | 3E-01 |
| IL6ST | -0.13 | 1E-01 | -0.06 | 7E-01 |
| KCNC1 | -0.11 | 2E-01 | 0.07 | 7E-01 |
| KLK6 | -0.09 | 3E-01 | -0.10 | 6E-01 |
| LY6H | -0.17 | 7E-02 | -0.14 | 4E-01 |
| MEGF10 | -0.21 | 2E-02 | -0.15 | 4E-01 |
| MOG | -0.11 | 2E-01 | 0.03 | 9E-01 |
| NCAN | -0.34 | 1E-04 | -0.17 | 3E-01 |
| NFASC | -0.21 | 2E-02 | 0.00 | 1E+00 |
| NPTX1 | -0.25 | 5E-03 | -0.34 | 5E-02 |
| NPTXR | -0.25 | 5E-03 | -0.22 | 2E-01 |
| NRCAM | -0.20 | 3E-02 | -0.17 | 3E-01 |
| NRGN | -0.32 | 3E-04 | 0.15 | 4E-01 |
| OMG | -0.31 | 4E-04 | -0.23 | 2E-01 |
| PAM | -0.16 | 8E-02 | -0.17 | 3E-01 |
| PDYN | -0.20 | 3E-02 | 0.06 | 7E-01 |
| PEBP1 | -0.19 | 4E-02 | -0.09 | 6E-01 |
| PIK3IP1 | -0.12 | 2E-01 | -0.03 | 9E-01 |
| POMC | -0.13 | 1E-01 | -0.22 | 2E-01 |
| PTAU | -0.28 | 2E-03 | 0.01 | 1E+00 |
| RPH3A | -0.27 | 2E-03 | -0.20 | 3E-01 |
| SELENOP | -0.02 | 8E-01 | 0.08 | 7E-01 |
| SEMA7A | -0.25 | 6E-03 | -0.27 | 1E-01 |
| SLITRK1 | -0.26 | 4E-03 | -0.12 | 5E-01 |
| SNCB | -0.21 | 2E-02 | -0.03 | 9E-01 |
| TMEM132D | -0.29 | 1E-03 | 0.02 | 9E-01 |
| TTAU | -0.28 | 2E-03 | 0.03 | 9E-01 |
| VASN | -0.13 | 1E-01 | -0.06 | 7E-01 |
| VCAM1 | -0.09 | 3E-01 | -0.08 | 6E-01 |
| VGF | -0.34 | 1E-04 | -0.26 | 1E-01 |
| VWC2L | -0.07 | 5E-01 | 0.12 | 5E-01 |
